# Mandibular Fractures in Edentulous Patients with Bone Atrophy and Osseointegrated Dental Implants: Therapeutic Management in a Case Series

**DOI:** 10.3390/medicina60111835

**Published:** 2024-11-08

**Authors:** Marta Benito Anguita, Jose Luis del Castillo Pardo de Vera, Saad Khayat, Ana María López López, Javier González Martín Moro, Gema Arenas de Frutos, Raúl Antúnez-Conde Hidalgo, Marta Pampín Martínez, Estela Gómez Larren, Carlos Navarro Cuéllar, Jose Luis Cebrián Carretero, Ignacio Navarro Cuéllar

**Affiliations:** 1 Department of Oral and Maxillofacial Surgery, Hospital General Universitario Gregorio Marañón, Universidad Complutense de Madrid, 28007 Madrid, Spain; maxilohgugm@gmail.com (M.B.A.); saad.khayat@gmail.com (S.K.); gema.arenas@gmail.com (G.A.d.F.); antunezconde_92@hotmail.com (R.A.-C.H.); nnavcu@hotmail.com (I.N.C.); 2 Department of Oral and Maxillofacial Surgery, Hospital Universitario La Paz, 28046 Madrid, Spain; jldelcastillopardo@gmail.com (J.L.d.C.P.d.V.); jgmartinmoro@salud.madrid.org (J.G.M.M.); mpampin@ucm.es (M.P.M.); josel.cebrian@salud.madrid.org (J.L.C.C.); 3 3D Printing Unit, Hospital General Universitario Gregorio Marañón, 28007 Madrid, Spain; egomezlarren.externo@salud.madrid.org

**Keywords:** mandible fracture, atrophic mandible, dental implants, rigid fixation, bone graft

## Abstract

*Background and Objectives:* This research describes the management of mandibular fractures in edentulous patients with atrophic mandibles and implant-retained overdentures, exploring etiologies and treatment options. *Materials and Methods*: A retrospective study (January 2010–December 2023) was conducted on six patients from two hospitals (Hospital Gregorio Marañón y Hospital La Paz, Madrid). The data collected included fracture etiology, treatment type, and complications. *Results*: All six patients were women, with a mean age of 76.33 years. The most common cause of fracture was peri-implantitis (50%). Surgical treatment (open reduction and internal fixation) was performed in five patients, with different surgical approaches and fixation methods. One patient, due to multiple comorbidities, received conservative treatment. Complications occurred in 50% of cases, including delayed healing and hypoesthesia. The average hospital stay was four days, with a mean follow-up of 34 months. *Conclusions*: Mandibular fractures in these patients are rare. Surgical treatment using rigid fixation plates is recommended. The rational use of bone grafting should be taken into account. Treatment depends on fracture type, patient condition, and surgeon experience.

## 1. Introduction

Mandibular atrophy is a consequence of tooth loss. Over time, edentulous bone decreases in height and also frequently in width. The remaining bone is characterized as being mostly cortical and less medullary. In addition, its periosteal vascular supply is partially diminished, and its endosteal vascularization may be severely compromised by the osseous tissue atrophy (the inferior alveolar artery can be found underneath the mucosa). The greatest degree of atrophy is usually located in the mandibular body [1].

Patients with mandibular atrophy can benefit from dental implant treatment. Subsequent to dental implant placement, one of the possible prosthetic solutions could be the use of implant-retained overdentures, with a success rate ranging from 80% to 100% [2,3,4]. Despite the high rate of osseointegration of the implants and the good functioning of overdentures, complications may arise. Infections, improper implant placement, bleeding, or mandibular fractures should be kept in mind when treating these patients. The incidence of mandibular fractures, in this specific situation, is very low (0.05–0.2%) [2,5,6,7], and few cases have been reported in the literature. The etiology of the fracture is usually due to trauma or peri-implantitis, and their consequences can be severe [5,8], including osteomyelitis, paresthesias/dysesthesias, pseudoarthrosis, and long-term nutritional problems [2].

The initial suspected diagnosis should be clinical, based on an accurate physical examination. It is important to highlight that bone fragment mobility may not be clear due to the splinting effect of the overdenture bar or the overdenture itself. Therefore, clinical examination should always be supported by imaging studies, ideally orthopantomography (OPG) and multiplanar computed tomography (CT).

The treatment of this type of fracture can be complex, and there are several different therapeutic alternatives, such as conservative management with a soft diet, splinting supported by implant-retained overdentures, or open reduction and internal fixation with or without combining the use of bone grafts [9,10]. Whether to choose one therapeutic option or the other depends primarily on the characteristics of the fracture, the possible associated peri-implantitis and the patient’s medical condition. In general, surgical treatment with an extraoral approach and rigid fixation is recommended, as it is a load-bearing situation where the osteosynthesis material must withstand all the biomechanical requirements of the fracture. In some cases, open reduction and internal fixation (ORIF) may be achieved by preserving dental implants if there is no indication for its removal, such as in cases where the cause of the fracture is peri-implantitis, or where there is implant mobility [9,11].

The main objective of this research is to describe the management of these types of fractures in a case series of edentulous patients with mandibular atrophy and implant-retained overdentures, as well as to discuss possible etiologies and treatment options.

## 2. Materials and Methods

A retrospective observational study of a case series was designed for the period from January 2010 to December 2023. The research was conducted in two different centers by the Oral and Maxillofacial Surgery Departments of Gregorio Marañón University Hospital (HGUGM) and La Paz University Hospital (HULP) in Madrid. A specific informed consent form was designed. This study is endorsed by the ethics committee of the Gregorio Marañón University Hospital.

### 2.1. Inclusion and Exclusion Criteria

The study included patients who met all the following criteria:

- Edentulous patients with atrophic bone treated with dental implants and implant-retained overdentures.
- Adult patients (over 18 years old).
- Patients with a clinical diagnosis of mandibular fracture supported by imaging evidence.
- Minimum clinical follow-up of one year.

Patients who met any of the following criteria were excluded from the study:

- Patients who have undergone previous surgeries that could have altered mandibular anatomy.
- Pathological mandibular fractures secondary to tumoral pathology.
- Patients with psychiatric disorders.
- Patients whose medical records did not include the study variables.
- Refusal to sign the informed consent.
- Patients with signs of parafunctional habits (bruxism).

The sources of information used to identify potential candidates for the research are as follows:

- Surgical reports from the Oral and Maxillofacial Surgery Department of HGUGM and HULP.
- Medical records from HGUGM and HULP.
- Radiological image archives from HGUGM and HULP.

### 2.2. Study Variables

The data collection notebook is an Excel table. The variables for which information was collected are as follows: sex, age, smoking habit, personal medical condition, history of radiation therapy in the head and neck area, diagnostic imaging test, fracture focus location, type of fracture line, reason for consultation, etiology, treatment (conservative vs. surgical), surgical approach, type of internal fixation, use of bone grafts, length of hospital stay, complications, and follow-up duration. A descriptive statistical analysis was performed.

### 2.3. Limitation of the Research

The primary limitations of the study are its retrospective design and the small sample size.

## 3. Results

The total number of patients who met the inclusion/exclusion criteria was six. All patients included in the study were female. The mean age was 76.33 years old. None of the patients were smokers; two patients (33.33%) had a history of osteoporosis treated with bisphosphonates; however, none of them had received radiation therapy in the head and neck area. The reason for consultation in all cases was mandibular pain and inflammation. Hypoesthesia was present in one patient. The imaging diagnostic tests used were OPG and CT, except for one case in which only OPG was used. The fracture focus was left parasymphysis in two patients, the left mandibular body in three patients, and one bifocal fracture (left body and right ramus). Fracture lines were simple in five patients (83.33%). The most frequent cause of fracture was peri-implantitis in three patients (50%); mandibular trauma in one patient (16.67%), and bisphosphonate-related osteonecrosis in two patients (33.33%). Among the patients with peri-implantitis, it is noteworthy that in one case, the fracture occurred at the moment of screwing the overdenture bar to the osseointegrated implant (Table 1).

Surgical treatment (open reduction and internal fixation) was performed in five patients (83.33%). A combined intraoral and cervical approach was used in two patients (40%), only a cervical approach in two patients (40%), and only an intraoral approach in the other patient (16.66%). Conservative treatment was employed in a 94-year-old patient with multiple pathologies who presented with a double mandibular fracture with non-displaced foci.

**Figure 1:**
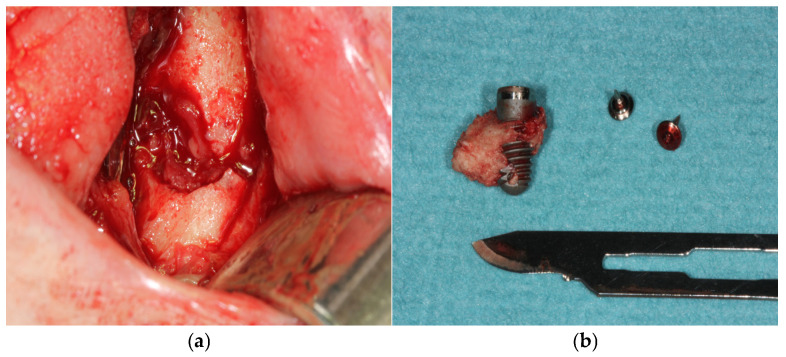
(**a**) Surgical defect after implant removal due to peri-implantitis. (**b**) Dental implant associated with necrotic bone after removal.

Internal fixation was performed using load-bearing principles. A 2.5 mm thick mandibular locking plate with a 2.4 mm screw diameter was used in three patients (50%), a combination of 2.0 mm thick mandibular locking plate with 2 mm screw diameter and 1.0 mm thick mandibular plate with 2 mm screw diameter was used in one patient (16.66%); and a 2.0 mm thick mandibular locking plate with 2 mm screw diameter combined with a titanium mesh in another patient (16.66%). Implant removal was necessary in two patients (33.33%) (Figure 1).

**Figure 2:**
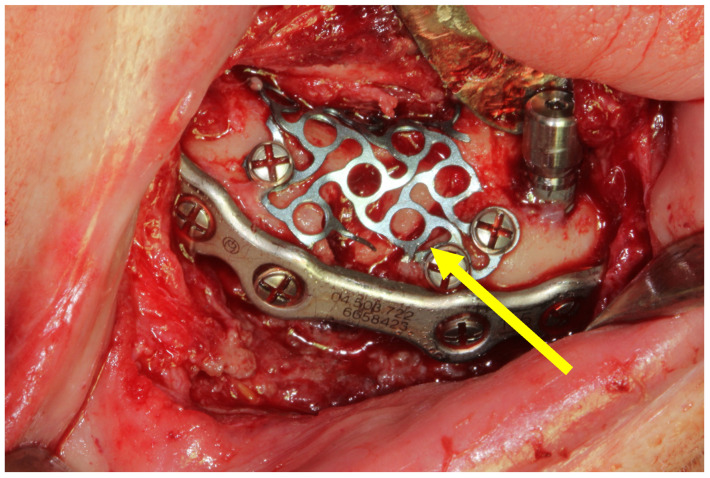
Open reduction and internal fixation of a mandibular fracture using a 2.0 mm thick mandibular locking plate with a 2 mm screw diameter. The yellow arrow points to the autologous bone graft obtained from the mandibular ramus and covered by a titanium mesh.

Additionally, in two patients (33.33%), autologous grafts obtained from the iliac crest and mandibular ramus were used, respectively (Figure 2). In one patient (16.66%), a non-autologous bone graft was used.

The average length of hospital stay was 4 days. The complication rate was 50% (three patients), with complications including delayed consolidation without interfragmentary mobility, hypoesthesia of the inferior alveolar nerve, and bone sequestration with granuloma formation, which was treated with antibiotic therapy. The follow-up period had a mean duration of 34 months. At the end of the follow-up period, all patients reported no pain.

### 3.1. Case 1

A non-smoker 70-year-old woman with a personal history of mitral valve disease consulted for pain and left paramandibular swelling. The patient denied any previous trauma. She had no history of radiation therapy in the head and neck area and had not received bisphosphonate treatment.

On physical examination, the patient had a complete edentulism with associated mandibular atrophy and carried a three-dental-implant-retained overdenture. No open mandibular fracture lines or interfragmentary mobility were observed.

**Figure 3:**
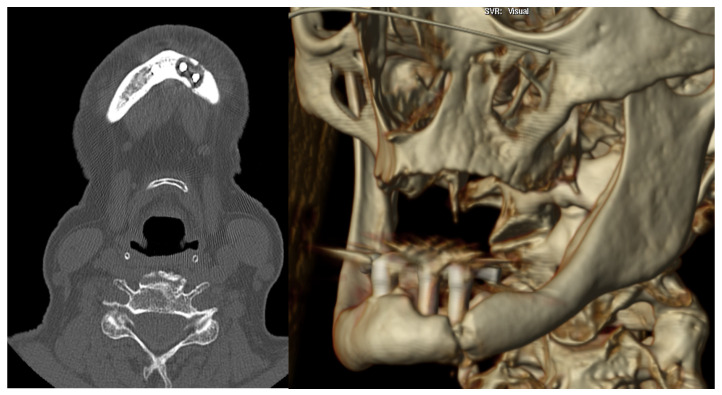
CT scan image: left mandibular parasymphisis fracture secondary to peri-implantitis.

**Figure 4:**
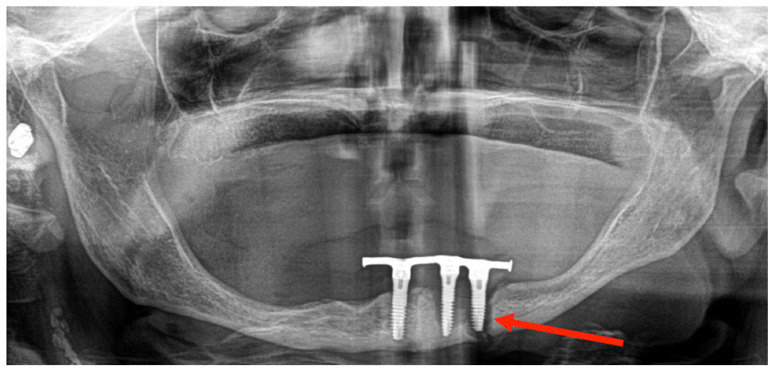
OPG image: left mandibular parasymphisis fracture secondary to peri-implantitis.

**Figure 5:**
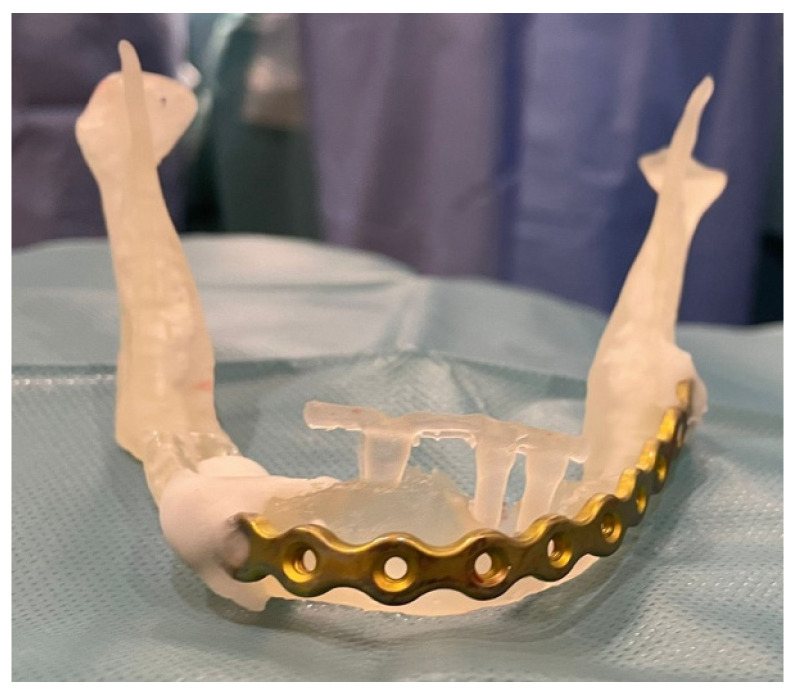
Three-dimensional biomodel with pre-adapted 2.5 mm thick mandibular locking plate with 2.4 mm screw diameter.

After performing an OPG and CT scan, she was diagnosed with a left mandibular body fracture due to peri-implantitis (Figure 3 and Figure 4). Based on the CT images, a 3D biomodel was created in the 3D Printing Unit (UPAM3D) of HGUGM. A 2.5 mm thick mandibular locking plate with a 2.4 mm screw diameter was preshaped on the 3D mandibular model before surgery (Figure 5).

**Figure 6:**
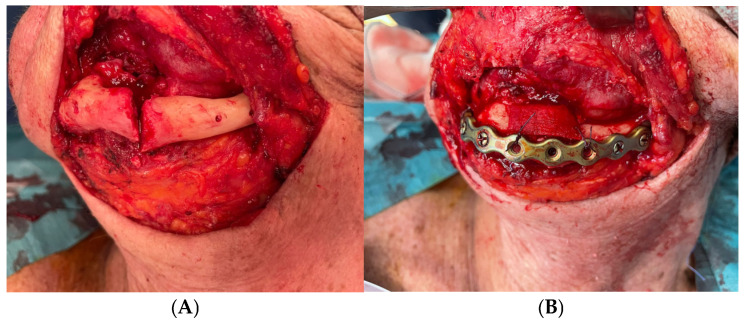
(**A**) External cervical approach appreciating a left parasymphysis fracture. (**B**) Open reduction and internal fixation with the prebended plate. Non-autologous bone graft was used.

Under general anesthesia and nasotracheal intubation, a cervical approach was used to identify the fracture and to perform an open reduction and internal rigid fixation with the previous prebended plate. Additionally, a non-autologous bone graft was used (Figure 6). Two implants affected by peri-implantitis were removed during the same surgical procedure, while the other implant was left in place to prevent further damage to the bone and its vascularization.

**Figure 7:**
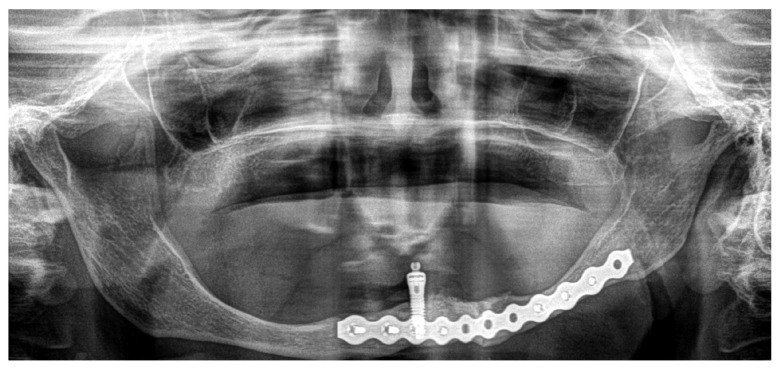
Postoperative OPG image demonstrates an adequate consolidation of the mandibular fracture.

**Figure 8:**
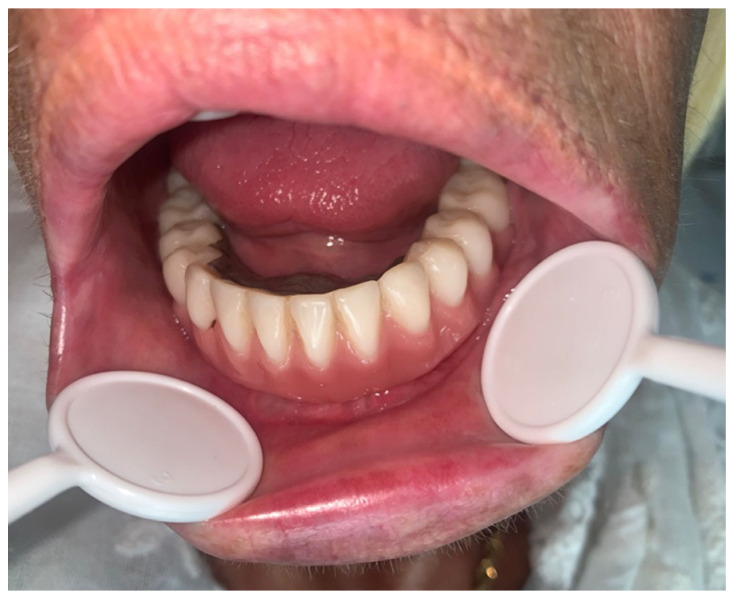
Postoperative intraoral appearance of the patient with one implant-retained overdenture.

During the immediate postoperative period, the patient had no medical events and was discharged on the first postoperative day. After 33 months, there were no clinical signs of complications, the fracture focus had consolidated (Figure 7), and the patient refused any kind of pre-prosthetic surgical intervention. For esthetic reasons, the patient carries one implant-retained overdenture (Figure 8).

### 3.2. Case 2

A 94-year-old woman with a medical history of heart failure who was a non-smoker and had no history of radiation therapy in the head and neck area consulted for mandibular pain following trauma after an accidental fall. The patient denied having received bisphosphonate treatment.

Physical examination revealed an edentulism and a mandibular atrophy. The patient carried a two-implant-retained overdenture as a dental prosthesis. No fracture lines or bone interfragmentary mobility were appreciated. The patient refers hypoesthesia of the left inferior alveolar nerve.

**Figure 9:**
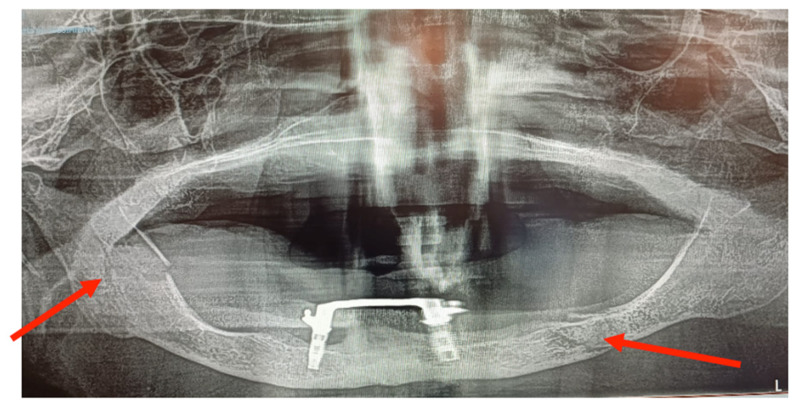
OPG image: bifocal atrophic mandibular fracture (red arrows point to fractures of the left mandibular body and the right mandibular ramus).

**Figure 10:**
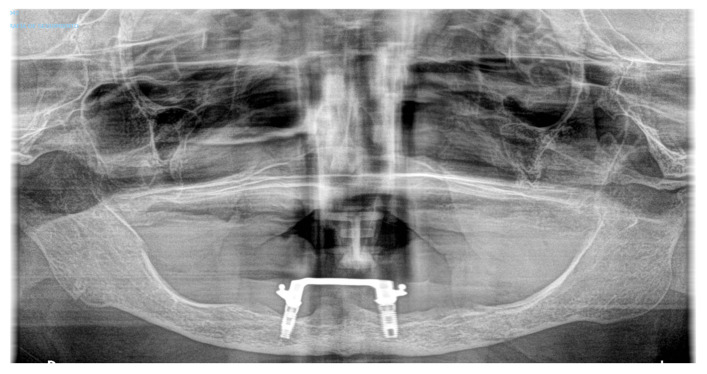
OPG image. Consolidated bifocal atrophic mandibular fracture (6 months post-injury).

The OPG image revealed a bifocal atrophic mandibular fracture (right mandibular ramus and left body) (Figure 9). Despite having two fracture foci, no important displacement was observed, and there was no pathological mobility between bone fragments. The patient refused surgery, so conservative treatment was carried out with a soft diet for 8 weeks. After close clinical follow-up for more than one year, it became evident that there was no important bone displacement or late complications (Figure 10), and the patient was able to return to wearing her overdenture.

## 4. Discussion

Edentulism causes the jawbone to atrophy, resulting in severe esthetic and functional consequences for chewing, management of the food bolus, and speech.

Implant placement and retained overdentures help edentulous patients [6,7,11] but do not prevent the progression of bone resorption, especially in the posterior area of the jaw. Therefore, in the event of infections, peri-implantitis, or trauma, the chances of suffering a mandibular fracture are higher [2].

Fractures of atrophic mandibles in patients carrying dental implants are rare, with an incidence reported in the literature ranging from 0.05% to 0.2% [2,5,6,7]. They are more common in women aged between 30 and 78 years old [2,9,12,13,14,15,16]. Some risk factors may include osteoporosis [11], previous radiation therapy in the head and neck area, use of anti-resorptive treatment, and smoking [8].

When considering the management of these fractures, it must be kept in mind that the alveolar process and the mandibular basal bone have been reduced [9], vascularization is impaired, and the bone morphology is predominantly cortical [13]. It seems that the placement of implants in these situations may result in an area of increased jaw weakness. Therefore, functional forces alone could cause a mandibular fracture without the need for trauma [6,12], and the more implants are placed, the greater the risk of mandibular fracture [11]. It is reasonable to understand that the surgical treatment of these patients also carries a higher risk of complications.

The treatment of these fractures can be a challenge for maxillofacial surgeons. There are different therapeutic options that are generally selected based on the characteristics of the involved fracture and the patient’s conditions. According to AO Foundation (Arbeitsgemeinschaft für Osteosynthesefragen) principles, they usually require surgical treatment with ORIF using rigid fixation plates due to the “load-bearing” situation, which implies that the osteosynthesis material must support the entire mandibular load [1].

The use of rigid versus semi-rigid fixation is a controversial issue well described in the literature. The rationale for using mini plates is that they require less periosteal stripping and thus less blood disruption, allowing for primary reduction and stability. However, the use of this type of plate is not always feasible in atrophic mandibles. Ellis et al. report that at least 10 mm of bone height is necessary to be able to accommodate two mini-plates. Even when this height is sufficient to fit two plates, the resulting stability is still lower than that observed in non-atrophic or dentate mandibles due to the fact that stability is directly influenced by the distance between plates [17]. It is also important not to forget that mini plates can be more susceptible to fractures [18]. Vajgel et al. [19] highlighted the need to establish safety limits for the fixation plates due to the variation in masticatory forces. Their research demonstrated that the application of forces of 102 N and 154 N to 1.0 mm and 1.5 mm plates, respectively, resulted in permanent deformation. In contrast, the 2.0 mm and 2.5 mm plates were deformed by higher forces, specifically 194 N and 260 N. Biomechanical studies indicate that a reduction in the vertical dimension of the mandible, particularly when the bone height at the fracture site is less than 10 mm, leads to a proportional decrease in resistance to bone fragment displacement. The absence of structural support in atrophic mandibles creates a scenario similar to a continuity defect. The result is that the bone along the fracture line does not bear any occlusal load, and most of the force is transferred to the plate. The development of locking systems has improved the treatment of these fractures, as the plate does not need to be in close contact with the underlying bone in all areas, and the vascularization is less compromised [20].

The cervical approach is the most commonly used [9] because it allows good bone exposure, permits the verification of a correct reduction of the fragments, and insets the appropriate osteosynthesis material. Another advantage of this approach in this type of fracture is having a better control of the inferior alveolar nerve. However, it has disadvantages such as the risk of facial nerve damage, development of cervical hematomas, infections, orocervical fistula, hypertrophic scars, and the exposure of osteosynthesis plates. ORIF can also be performed through an intraoral approach, communicating the oral cavity with the fracture site and osteosynthesis plates, thus increasing the risk of infection. However, there is no evidence confirming the superiority of one approach over another for atrophic mandibular fractures in patients with implants and overdentures [2]. Therefore, the choice of approach should also be based on each surgeon’s experience [12].

There is no convincing evidence that the use of bone grafting is necessary in the treatment of atrophic edentulous mandibular fractures. The use of bone grafting in these specific situations is useful to facilitate bone union, provide fracture stability and increase bone volume in order to prevent pathological fractures and improve prosthetic rehabilitation [17]. Autologous or non-autologous bone grafts can be employed in regions where there are significant bone defects or in clinical scenarios characterized by reduced healing capacity. On one hand, autologous grafts, particularly those harvested from the iliac crest, are frequently favoured due to their superior capacity to promote neovascularization. This biological process is crucial, as it enhances graft integration and survival at the recipient site, thereby facilitating better healing outcomes. Additionally, autologous grafts contain vital growth factors that contribute to the regenerative process, making them a reliable choice for complex cases. On the other hand, non-autologous grafts may be selected for their relative availability and the reduced morbidity associated with donor sites. It is important to highlight that the integration of non-autologous grafts may be less predictable, and their biological behaviour may not replicate that of autologous grafts. The preference between autologous and non-autologous grafts is influenced by a variety of clinical and practical factors, such as the extent of the bone defect, the patient’s general condition, and the surgeon’s familiarity with specific materials and techniques. 

Joshep E. et al. report that, despite the traditional view that the use of bone grafting is essential to enhance osteogenesis, their findings suggest that the use of large reconstruction plates may be sufficient to achieve adequate ossification without the need for grafting. This perspective challenges established paradigms and indicates that, with appropriate surgical techniques and materials, effective results can be achieved even in cases traditionally considered complex [21]. Luhr et al. achieved excellent results with open reduction and internal fixation with bone plates without the routine addition of bone grafts [22].

In certain circumstances, like non-displaced simple fractures without interfragmentary mobility, patients with significant comorbidities who are not candidates for surgical treatment, or patients unwilling to undergo surgery, conservative treatment may be a valid option. A soft diet and/or closed reduction with external fixation using the patient’s overdenture [10], or wires between the remaining implants [2,11,13] can help to obtain proper healing of the fracture.

New technologies allow for more predictable and precise procedures. The use of biomodels, pre-adapted plates, and personal–specific implants facilitates more detailed and personalized surgical planning. These innovations significantly reduce surgical time by enabling faster and more accurate plate adaptation. As a result, a decrease in the length of hospital stay can be observed.

## 5. Conclusions

Mandibular fractures in edentulous patients carrying implant-retained overdentures are a very rare condition. Consequently, there is no highly scientific consensus regarding their treatment.

In most cases, surgical treatment is preferred and requires the use of rigid fixation plates. Occasionally, bone grafting may be necessary.

Clinical features of the patient, the type of fracture, and the surgeon’s experience should be considered to select an appropriate treatment option.

## Figures and Tables

**Table 1:** Study variables.

Variable	Category	Total (% or Mean)
Age (years)		76.33
Gender	Male	0 (0%)
	Female	6 (100%)
Tobacco	Yes	0 (0%)
	No	6 (100%)
Osteoporosis treated with bisphosphonates	Yes	2 (33.3%)
	No	4 (66.7%)
Radiotherapy for head and neck	Yes	0 (0%)
	No	6 (100%)
Symptoms	Pain	6 (100%)
	Inflammation	6 (100%)
	Hypoesthesia	1 (16.67%)
Diagnostic imaging studies	Orthopantomography (OPG)	6 (100%)
	Computed tomography (TC)	5 (83.3%)
Location	Left parasymphyseal	2 (33.3%)
	Left body	3 (50%)
	Bifocal (left body and right ramus)	1 (16.67%)
Fracture pattern	Simple	5 (83.3%)
	Bifocal	1 (16.67%)
Etiology	Peri-implantitis	3 (50%)
	Traumatism	1 (16.67%)
	Osteonecrosis	2 (33.3%)
Treatment	Conservative	1 (16.67%)
	Surgical	5 (83.3%)
Surgical approach	Combined	2 (33.3%)
	Cervical	2 (33.3%)
	Intraoral	1 (16.67%)
Type of internal fixation	2.5 mm thick mandibular locking plate with 2.4 mm screw diameter	3 (50%)
	2.0 mm thick mandibular locking plate with 2 mm screw diameter + 1.0 mm thick mandibular plate with 2 mm screw diameter	1 (16.67%)
	2.0 mm thick mandibular locking plate with 2 mm screw diameter + titanium mesh	1 (16.67%)
Use of bone grafts	Non-bone graft	3 (50%)
	Autologous graft (iliac crest)	1 (16.67%)
	Autologous graft (mandibular ramus)	1 (16.67%)
	Non-autologous graft	1 (16.67%)
Implants removal	Yes	2 (33.33%)
	No	4 (66.7%)
Length of hospital stay (days)		4
Complications	No complication	3 (50%)
	Delayed consolidation	1 (16.67%)
	Hypoesthesia of the inferior alveolar nerve	1 (16.67%)
	Bone sequestration	1 (16.67%)
Follow-up duration (months)		34

## Data Availability

The data presented in this study are available upon request from the corresponding author. The data are not publicly available due to local policies.
